# 2607. The impact of portable high efficiency Hepa Filters on the incidence of invasive fungal infection in Covid 19 inpatients in a tertiary care hospital: a retrospective analysis from Pakistan

**DOI:** 10.1093/ofid/ofad500.2221

**Published:** 2023-11-27

**Authors:** A N E E R A AHMED, B U S H R A JAMIL, A I M A N SULTAN, S H O B A LUXMI

**Affiliations:** NICVD, KARACHI, Sindh, Pakistan; AKUH, KARACHI, Sindh, Pakistan; AKUH, KARACHI, Sindh, Pakistan; NICVD, KARACHI, Sindh, Pakistan

## Abstract

**Background:**

**Objective:** To identify impact of air filtration on incidence of Invasive fungal infections in COVID-19 patients after restructuring of COVID-19 wards.

**Introduction:**

Most COVID-19 patients present with mild or moderate disease; but patients with comorbidities may be predisposed to secondary and opportunistic infections. *Aspergillus* infections resulting from failure in air filtration systems have been reported. Our study aims to determine the impact of installation of such preventative measures in covid units on the frequency of invasive fungal disease as a complication of covid 19 illness

**Methods:**

All COVID-19 patients >18 years (non-severe, severe and/or critical) admitted at the tertiary care center were included. Even COVID-19 patients with suspected IPA, based on isolation of fungal species from one or more respiratory samples were included. Two time frames were used for inclusion of patients, one three months prior to and second three months after installation of HEPA filters in COVID wards. Patients with underlying respiratory disease, previous history of invasive pulmonary aspergillosis, on chronic steroid use ( >3 months), on broad-spectrum antibiotics and with hematological malignancies were excluded. SPSS version 23.0 was used for data analysis.

**Results:**

From 187 patients, 97 enrolled before installation of HEPA filters while 90 after installation of HEPA filters. Overall the mean age of patients was 62.7 ± 14.6 years. A significant difference of p-0.04 was observed in terms of length of stay in-between before installation of HEPA filter group (13.1 ± 11.2 days) and in after installation of HEPA filter group (10.2 ± 8.2 days). Beta-D-Glucan test with HEPA filter was positive in 17 (17.5%) of patients while in 28 (31.1%) of patients without HEPA filter (p-0.03). Treatment with Azoles was found to have significant difference in-between both groups (p-0.002). Overall, treatment was successful in 16 (16.5%) patients in without HEPA filter group while in 38 (42.2%) of patients with HEPA filter (p< 0.001)

**Conclusion:**

Substantial decrease in incidence of invasive fungal infections among COVID-19 patients was reported in terms of age, length of stay, Beta-D-Glucan, treatment with azoles and overall treatment in favor of HEPA filters.

**Disclosures:**

**All Authors**: No reported disclosures

